# Length of Hospitalization-Related Differences and Associated Long-Term Prognosis of Patients with Cardiac Resynchronization Therapy: A Propensity Score-Matched Cohort

**DOI:** 10.3390/jcdd9100354

**Published:** 2022-10-15

**Authors:** Yu Yu, Hao Huang, Sijing Cheng, Yu Deng, Chi Cai, Min Gu, Xuhua Chen, Hongxia Niu, Wei Hua

**Affiliations:** Cardiac Arrhythmia Center, State Key Laboratory of Cardiovascular Disease, Fuwai Hospital, National Center for Cardiovascular Diseases, Chinese Academy of Medical Sciences and Peking Union Medical College, Beijing 100037, China

**Keywords:** cardiac resynchronization therapy, lengths of hospitalization, death, heart failure, long-term prognosis

## Abstract

Previous studies indicated that prolonged lengths of hospitalization (LOH) during cardiac resynchronization therapy (CRT) implantation are associated with poorer physical status and higher in-hospital mortality. However, evidence on the impact of LOH on the long-term prognosis of CRT patients is limited. The purpose of this study was to assess LOH-related prognostic differences in CRT patients. In the propensity score-matched cohort, patients with standard LOH (≤7 days, n = 172) were compared with those with prolonged LOH (>7 days, n = 172) for cardiac function and study outcomes during follow-up. The study outcomes were all-cause death and heart failure (HF) hospitalization. In addition, cardiac function and changes in cardiac function at the follow-up period were used for comparison. At a mean follow-up of 3.36 years, patients with prolonged LOH, as compared with those with standard LOH, were associated with a significantly higher risk of all-cause death (hazard ratio [HR] 1.87, 95% confidence interval [CI] 1.18–2.96, *p* = 0.007), and a higher risk of HF hospitalization (HR 1.68, 95% CI 1.08–2.63, *p* = 0.023). Moreover, patients with standard LOH had a more significant improvement in cardiac function and a pronounced reduction in QRS duration during follow-up than those with prolonged LOH. LOH-associated differences were found in the long-term prognosis of CRT patients. Patients with prolonged LOH had a worse prognosis than those with standard LOH.

## 1. Introduction

Due to the high incidence and increasing prevalence of heart failure (HF) [1,2], the number of patients suitable for cardiac resynchronization therapy (CRT) implantation is rising yearly [3]. In this case, HF patients treated with CRT were more likely to have poorer physical status and comorbidities than those treated with medication [4]. Hospitalization of CRT candidates is a complex process, which intuitively reflects marked differences in length of hospitalization (LOH) during CRT implantation at our center. Prior to our study, several studies have shown that the LOH during CRT implantation varies between medical institutions, possibly due to differences in operator experience and hospital treatment patterns [5]. Nevertheless, this explanation does not reasonably account for the difference in LOH during CRT implantation in our center.

In addition, prolonged LOH forces patients with CRT to bear significantly higher hospital costs [6]. Apart from the cost of hospitalization, the LOH is also related to the patient’s physical condition during hospitalization. For instance, studies have shown differences in in-hospital mortality among patients with various LOH during CRT implantation [7]. Milner, A. et al. [8] patients with a higher frailty index had a markedly higher LOH during CRT implantation. However, this limited evidence can only infer that a longer LOH during CRT implantation is associated with poorer inpatient physical status. Of note, these issues have prompted the investigators to further consider whether the LOH during CRT implantation may have an impact on the long-term prognosis of such patients. Regrettably, the previous study failed to provide evidence to answer the above research hypothesis.

Therefore, this study was designed to address two aims to verify the proposed hypothesis. First, to explore the differences in cardiac function among patients with different LOH during CRT implantation. Second, our objective was to assess whether there are LOH-related differences in long-term prognosis among patients with CRT.

## 2. Materials and Methods

### 2.1. Study Population

**Figure 1:**
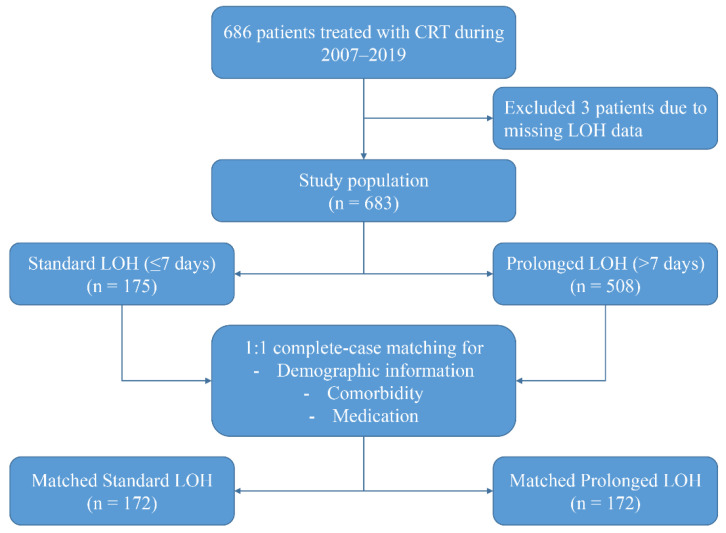
Study Population.

This study was a single-center, retrospective, observational cohort study. A total of 686 patients with HF who underwent CRT implantation at the Arrhythmia Center, FuWai Hospital, were consecutively enrolled in this study between March 2007 and March 2019. All patients receiving CRT implantation fulfilled the guideline-recommended Class I or Class II indications [3]. After excluding samples with missing LOH data and other important information, a total of 683 patients were entered into the final analysis (Figure 1). This study was conducted in accordance with the Declaration of Helsinki and was approved by the Ethics Committee of FuWai Hospital, Chinese Academy of Medical Sciences (IRB2012-BG-006). All patients included in the study signed an informed consent form.

### 2.2. Data Collection

The baseline patient characteristics are derived from the electronic medical record, including basic inpatient information, surgical records, and discharge summaries. The details of CRT implantation have been described in previous studies [9]. Demographic information (age, gender) was obtained by questionnaire. History of disease and medication was collected from the patient’s electronic medical record. Disease history included left bundle branch block (LBBB), hypertension, diabetes, coronary artery disease (CAD), ventricular tachycardia/ventricular fibrillation (VT/VF), atrioventricular block (AVB), atrial fibrillation (AF), and cardiomyopathy, Medication history includes the presence of inpatient medications and post-discharge medications, consisting of angiotensin-converting enzyme inhibitor/angiotensin receptor blocker (ACEI/ARB), β-blocker, spironolactone (Spiro), digoxin, diuretics statin, and amiodarone. BMI was calculated as weight (kg) divided by height squared (m2). NYHA cardiac function classification was determined by the clinician based on the patient’s symptoms, medical history, and clinical tests and examinations of cardiac structure and function. Left ventricular ejection fraction (LVEF), left ventricular end-diastolic diameter (LVEDD), CRT-defibrillator (CRT-D), biventricular pacing (BVP) proportion, QRS duration, N-terminal prohormone of brain natriuretic peptide (NT-proBNP), and estimated glomerular filtration rate (eGFR) were collected using electronic medical records. The eGFR (mL/min/1.73 m^2^) was calculated by the simplified MDRD formula [10]. 

The LOH began on the day of admission, ended at patient discharge during the same hospitalization, and was mainly composed of pre-operative preparation time, surgery time, and post-operative hospitalization time. At our medical center, CRT patients are usually discharged within two days after CRT implantation, and their standard LOH is approximately five days. However, some patients happen to have surgery on a Friday, and since discharge is rarely processed on the weekend, they are usually discharged on the following Monday. Due to the above reasons, this study classified ≤7 days as the standard LOH for CRT patients, and more than 7 days as a prolonged LOH. All baseline information was obtained during CRT implantation.

### 2.3. Study Outcomes and Follow-Up

The study outcome were all-cause death and heart failure (HF) hospitalization. The follow-up deadline was January 2021. Follow-up for study outcomes was from CRT implantation until the date of first HF rehospitalization or death. Information on study outcomes was collected through hospital outpatient records and medical telephone calls. In addition to assessing study outcomes, we also evaluated cardiac function, including LVEF, LVEDD, NT-proBNP, and QRS duration, at 6 months after CRT implantation. Changes in cardiac function were defined as the difference between cardiac function parameters during follow-up minus baseline.

### 2.4. Statistical Analysis

**Figure 2:**
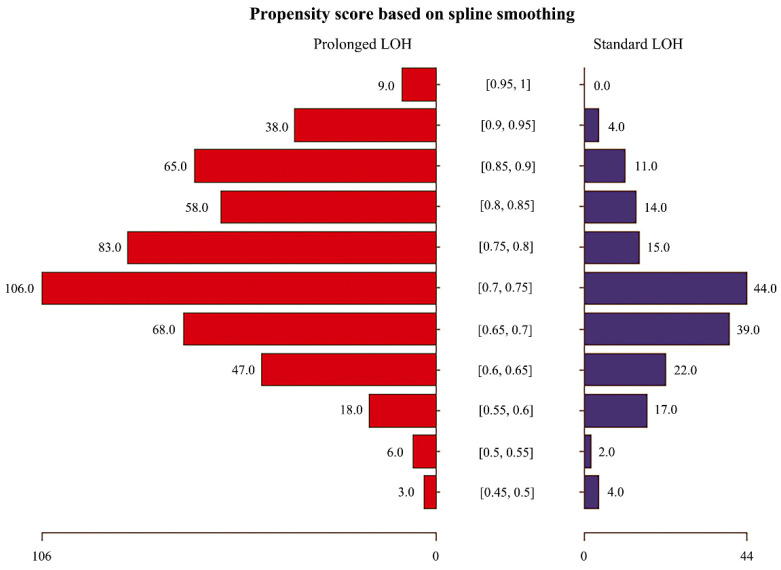
The distribution of propensity scores in the matched cohort.

Given the potential differences in baseline characteristics between the two groups of eligible participants, propensity score matching was performed to identify a cohort of patients with similar baseline characteristics (Table 1). Matching was performed using a 1:1 matching protocol without replacement (greedy-matching algorithm), and the matching tolerance value was 0.05 [11]. The baseline characteristics used for propensity score matching included: demographic information (age, gender), comorbidities, and medication history. Standardized differences were used to estimate the balance of the matched baseline covariates, with standardized differences less than 10.0% indicating relatively small imbalances [12]. Figure 1 shows the study population’s selection process, including the population screening and the construction of the matching cohort. Figure 2 and the Supplementary Material illustrate the distribution of propensity scores in the matched cohort and the corresponding propensity scores for each matched pair of patients. 

In the matched cohort, continuous variables are presented as (N) Mean ± standard deviation (SD), and categorical variables are presented as N (%). McNemar’s test was used for categorical variables, and Student’s *t*-test or paired sample test was used for continuous variables for paired comparisons. Comparative risks for study outcomes were assessed using the Cox proportional-hazards regression model and Kaplan–Meier (KM) survival analysis. To better achieve a balanced distribution of baseline characteristics between groups, we utilized Genetic Matching (GenMatch) for subgroup analysis. GenMatch matching is an extension of the propensity score and Mahalanobis distance matching, which maximizes the observed differences in outcome variables between groups through repeated matching [13,14]. A two-sided *p*  <  0.05 was considered significant. All statistical analyses were performed using the R programming language (version 4.0.2) and GraphPad Prism 9.

## 3. Results

### 3.1. Baseline Characteristics

Before propensity score matching, there were differences between the two groups in several baseline variables (Table 1). With propensity score matching, 172 patients with standard LOH (≤7 days) during CRT implantation were matched to 172 patients with prolonged LOH (>7 days). In the propensity score-matched cohort, patients with prolonged LOH had worse cardiac function compared to those with standard LOH (NYHA IV: 6.6% vs. 17.1%; LVEDD: 68.01 ± 9.15 mm vs. 69.98 ± 10.30 mm; NT-proBNP: 1710.70 ± 1733.10 pg/mL vs. 2027.64 ± 2917.72 pg/mL) and higher incidence of VT/VF and AF (VT/VF: 19.2% vs. 30.2%; AF: 14% vs. 23.3%), with a standardized difference significantly higher than 10.0%. More details on comparing baseline characteristics between groups are shown in Table 1.

### 3.2. Cardiac Function and Study Outcomes during Follow-Up

**Figure 3:**
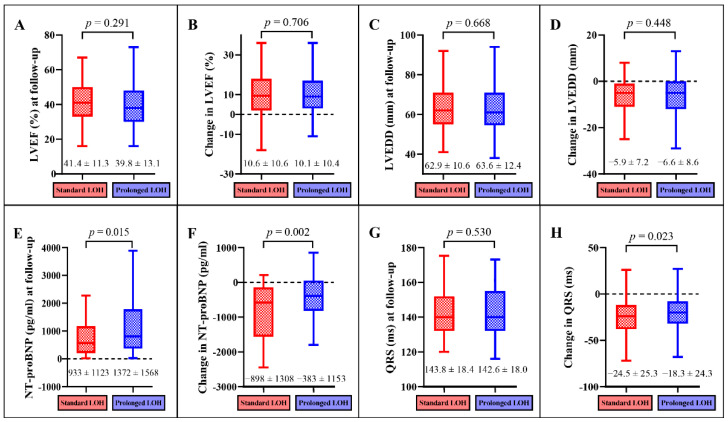
Comparison of cardiac function and changes in cardiac function during follow-up. Comparison of LVEF at follow-up (**A**), change in LVEF (**B**), LVEDD at follow-up (**C**), change in LVEDD (**D**), NT-proBNP at follow-up (**E**), change in NT-proBNP (**F**), QRS at follow up (**G**), and change in QRS (**H**).

The comparison of follow-up results between the standard LOH and prolonged LOH groups in the propensity score-matched cohort are displayed in Figure 3. Improvements in cardiac function were observed during the follow-up period in CRT patients with standard LOH and those with prolonged LOH. Compared with patients with prolonged LOH, patients with standard LOH had lower NT-proBNP levels (933 ± 1123 pg/mL vs. 1372 ± 1568 pg/mL, *p* = 0.015, Figure 3E), a more significant reduction in NT-proBNP (−898 ± 1308 pg/mL vs. −383 ± 1153 pg/mL, *p* = 0.002, Figure 3F), and a more significant reduction in QRS duration (−24.5 ± 25.3 ms vs. −18.3 ± 24.3 ms, *p* = 0.023, Figure 3H). 

**Figure 4:**
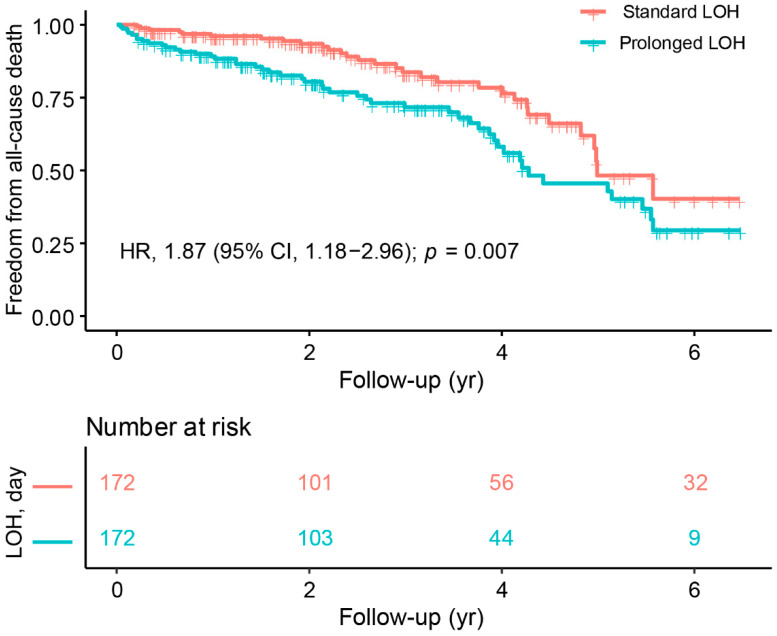
Kaplan–Meier survival curves of freedom from all-cause death.

**Figure 5:**
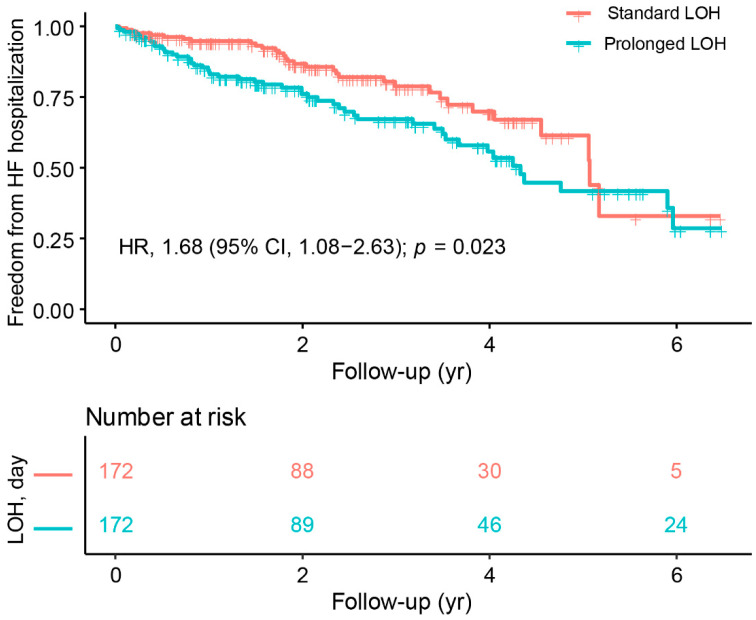
Kaplan–Meier survival curves of freedom from HF hospitalization.

In terms of outcome, patients with prolonged LOH had a higher incidence of all-cause death (18.2% vs. 29.7%, *p* = 0.019) and a higher incidence of HF hospitalization (19.2% vs. 29.8%, *p* = 0.032) than those with standard LOH. Figure 4 and Figure 5 display the differences in study outcomes associated with LOH in CRT patients at a mean follow-up of 3.34 years. The KM curve and Cox regression results showed that patients with prolonged LOH had a significantly higher risk of all-cause death than those with standard LOH (HR 1.87, 95% CI 1.18–2.96, *p* = 0.007) (Figure 4). Similarly, patients with prolonged LOH had a higher risk of HF hospitalization than those with standard LOH (HR 1.68, 95% CI 1.08–2.63, *p* = 0.023) (Figure 5). 

In addition, we conducted a survival analysis according to three different follow-up periods: 0–2 years, 2–4 years, and 4–6 years. During the 2-year follow-up period, patients with prolonged length of hospitalization (LOH) had a significantly higher risk of all-cause mortality compared with those with standard LOH (HR, 3.16; 95% CI, 1.46–6.82; *p* = 0.004). However, as the follow-up period was extended, there was no significant difference in the risk of mortality between the standard LOH and prolonged LOH groups during 2–4 years (HR, 1.33; 95% CI, 0.50–2.53; *p* = 0.767) and 4–6 years (HR, 1.13; 95% CI, 0.41–2.45; *p* = 0.891). Similarly, patients with prolonged LOH had a significantly higher risk of heart failure (HF) hospitalization than those with standard LOH during the 2-year follow-up period (HR, 2.13; 95% CI, 1.17–3.88; *p* = 0.014). However, with increasing follow-up, there was no significant difference in the risk of HF hospitalization between the standard and prolonged LOH groups during 2–4 years (HR, 1.57; 95% CI, 0.68–3.60; *p* = 0.288) and 4–6 years (HR, 0.87; 95% CI, 0.27–2.82; *p* = 0.818).

### 3.3. Sensitivity Analysis Using GenMatch Matching

We used GenMatch matching to demonstrate the results’ stability and to observe LOH-related differences in study outcomes to the greatest extent possible. In the GenMatch-matched cohort, as shown in Table 2, patients with prolonged LOH had lower LVEF values, higher values of LVEDD and NT-proBNP, less change in QRS duration, and a higher incidence of all-cause death and HF hospitalization during follow-up than those with standard LOH (all *p* < 0.05). 

### 3.4. Changes in LOH across Surgery Years

**Figure 6:**
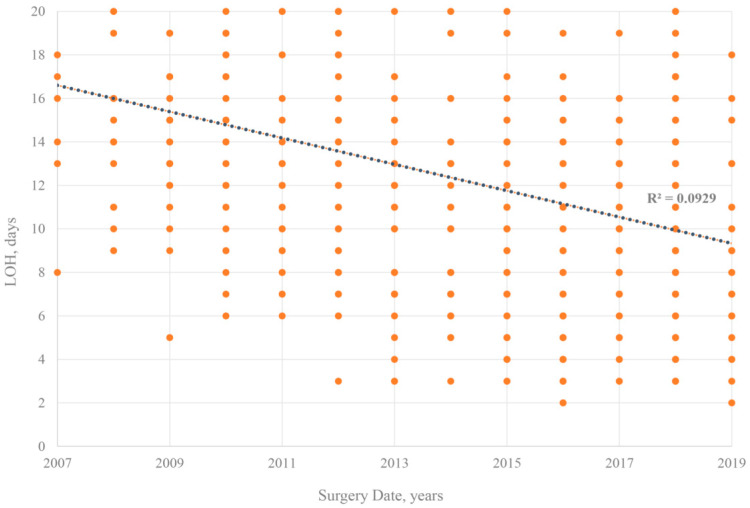
Changes in LOH across surgery years.

In addition, given that with the improvement of CRT technology, there may be a corresponding decline in LOH. Pearson’s correlation coefficient was used to examine potential associations between the study variables. As shown in Figure 6, the negative connection was not significant (R^2^ = 0.093).

## 4. Discussion

In a large sample of propensity score-matched cohorts, we compared baseline cardiac function and long-term prognosis in patients with different LOH during CRT implantation. The main findings are as follows. First, the prolonged LOH during CRT implantation may reflect poorer cardiac function in hospitalized patients. Second, we observed that patients with standard LOH and prolonged LOH could obtain an improvement in cardiac function from CRT treatment at long-term follow-up. More importantly, patients with standard LOH experienced a more pronounced improvement in cardiac function and a significant reduction in QRS duration during the follow-up period than those with prolonged LOH. Third, the results of the Cox regression analysis and KM curves supported the LOH-related difference in long-term prognosis that patients with standard LOH had a better prognosis than those with prolonged LOH. Fourth, with GenMatch matching, patients with standard LOH experienced better cardiac function, more significant cardiac function improvements, and a lower incidence of all-cause death and HF hospitalizations than those with prolonged LOH during follow-up.

Prior to our study, several studies had begun to focus on the clinical value of LOH. Banks, H. et al. [7] reported that patients with different LOH during pacemaker implantation had inconsistent in-hospital mortality. Milner, A. et al. [8] found that the prolonged LOH was likely influenced by the poorer physical condition of the hospitalized patients. In addition, Daghistani, T.A. et al. [15] developed a predictive model to predict the LOH in cardiac patients and found that factors affecting the LOH included age, heart rate, blood pressure, and diabetes. However, the exploration of LOH was limited to the period of CRT implantation and before hospitalization, and failed to investigate the relationship between LOH and long-term outcomes in CRT patients. This may be since the researchers believe that LOH exerts a limited impact on prognosis. Unlike previous studies, our results not only support that LOH is a reliable indicator for assessing cardiac function during CRT implantation, but that further results suggest that this indicator is significantly associated with long-term prognosis.

Notably, patients with prolonged LOH in this study are easily misclassified as “CRT non-responders”. A general consensus on the definition of “CRT non-response” is that echocardiographic parameters such as LVEF and left ventricular diameter failed to improve or even worsened at 6 months after CRT implantation [16,17]. Apparently, neither patients with standard nor prolonged LOH in our study met the definition of CRT non-response, because these patients observed an improvement in cardiac function during the follow-up period. Patients with standard LOH experienced more significant improvements in cardiac function and were at lower risk of death and HF re-hospitalization than those with prolonged LOH. Our findings suggest that even for those patients who benefit from CRT, there is still a LOH-related difference in their prognosis. According to the above findings, this study proposes that clinicians can promptly classify CRT patients as likely to have a better prognosis and a worse prognosis based on LOH after discharge. For patients with markedly prolonged LOH, clinicians need to pay more attention to these patients and make their best efforts to improve the prognosis as much as possible.

In our study, almost 3/4 of the patients had a significantly longer LOH during CRT implantation. We found that patients with prolonged LOH had a worse cardiac function and significantly higher incidence of VT/VF and AF. For such patients, the clinician has to maintain their hemodynamic stability in addition to performing CRT implantation. In fact, as a national cardiovascular center, patients who visit our hospital have worse cardiac function compared to a general medical center, especially for CRT candidates. For the reasons mentioned above, the CRT patients at our medical center have a longer LOH because these patients need a longer LOH for appropriate treatment. Of note, the interesting point in our study is that the shorter LOH reflects a more significant reduction in the QRS duration. Patients with a shorter QRS duration had greater responsiveness to CRT, which contributed to a more pronounced improvement in their prognosis. Our findings suggest that clinicians may be able to evaluate the degree of reduction in QRS duration by means of LOH, which could potentially help in prognostic improvement.

In the present study, we not only observed changes in cardiac remodeling parameters during follow-up, but also performed longer follow-ups to investigate LOH-related differences in outcome events. As expected, patients who experienced prolonged LOH during CRT implantation had a smaller improvement in cardiac remodeling parameters and a higher risk of adverse events during follow-up. Several reasons can be used to explain this phenomenon. First, patients with prolonged LOH had a higher proportion of NHYA IV, higher levels of BT-proBNP, and a higher incidence of VT/VF and AF at baseline. This reflects that patients with prolonged LOH experienced worse cardiac function than those with standard LOH during CRT implantation. For patients with poor cardiac function, clinicians usually perform an adequate pre-operative evaluation [18], as well as a longer post-operative period to observe the effect of CRT, and these objective factors contribute to the prolonged LOH. In addition, we observed that the increased duration of pre-operative examinations resulted in a significant prolongation of LOH in our medical center. This means that these patients have relatively inadequate pre-operative information, thereby reflecting that they usually pay less attention to their health status. Thus, patient neglect of their health status is also an important factor to consider.

Regardless of the effect of LOH, our findings encourage that patients eligible for CRT implantation should receive CRT, which contributes to an improvement in cardiac function, especially for those patients with better cardiac function at baseline. Furthermore, clinicians can easily identify CRT patients with poor prognoses after discharge with the help of LOH. More importantly, clinicians should pay more attention to patients with prolonged LOH and identify risk factors affecting prognosis as soon as possible to improve prognosis. On the other hand, despite propensity score matching in Table 1, patients with prolonged LOH had higher rates of VT/VF, AF, amiodarone, and NYHA III-IV, suggesting that these patients had relatively poorer cardiac structure and function. For this group of patients, it appears that AF and VT/VF significantly worsen their prognosis, which can hamper their clinical benefit from CRT despite the high rate of amiodarone use. Recently, the APAF-CRT mortality trial [19] suggested that the ablation + CRT was superior to pharmacological therapy in reducing mortality in HF patients with AF. Therefore, for patients with significantly prolonged LOH and severe arrhythmias, using new treatment strategies may help improve the prognosis.

Some limitations of this study should be mentioned. First, this was a non-randomized, observational study, although propensity score matching was performed robustly. However, potential selection bias should be noted [20]. For example, our study population may involve some patients from emergency clinics, and emergency patients’ examination times may be rushed, resulting in a marked reduction in LOH. Second, the selection bias of retrospective studies cannot be avoided. Therefore, our study finds that more studies are needed in the future. Third, the LOH in this study failed to further break out the duration of surgery, pre-operative time, and post-operative time. Information on these details may help to provide insight into the relationship between LOH and prognosis of patients with CRT. Fourth, as the CRT implantation technique improved, there was a corresponding decline in LOH, but this connection was not significant (R^2^ = 0.09). It also indicates that the results of this study are stable and not easily influenced by selection bias.

## 5. Conclusions

Our findings provide evidence that LOH-related differences in improvements in cardiac function and freedom from death and HF re-hospitalization in CRT patients. Patients with prolonged LOH experienced a minor improvement in cardiac function and a reduction in QRS duration than those with standard LOH. Furthermore, patients with prolonged LOH had a significantly increased risk of adverse outcomes. The conclusion of this study supports that LOH can be a reliable predictor of cardiac function and long-term prognosis in patients with CRT.

## Figures and Tables

**Table 1:** Baseline characteristics before and after propensity score matching.

Characteristic	Before Matching			After Matching		
	Standard LOH (n = 175)	Prolonged LOH (n = 508)	Standardized Difference (%)	Standard LOH (n = 172)	Prolonged LOH (n = 172)	Standardized Difference (%)
LOH, day	5.59 ± 1.47	14.15 ± 5.93	198.0	5.60 ± 1.47	14.03 ± 5.40	213.0
Age, y	58.64 ± 11.33	59.23 ± 11.58	5.0	58.57 ± 11.39	59.39 ± 11.22	7.2
Male, n (%)	118 (67.43)	347 (68.31)	2.0	116 (67.4)	115 (66.9)	1.2
BMI, kg/m^2^	24.84 ± 4.97	24.37 ± 3.69	11.0	24.84 ± 4.97	24.25 ± 3.63	13.6
Comorbidity, n (%)						
LBBB	137 (78.74)	364 (71.94)	16.0	137 (79.7)	120 (69.8)	18.9
Hypertension	59 (33.91)	186 (36.69)	6.0	58 (33.7)	68 (39.5)	9.1
Diabetes	46 (26.44)	123 (24.26)	5.0	45 (26.2)	39 (22.7)	8.1
CAD	49 (28.16)	143 (28.21)	0.0	49 (28.5)	47 (27.3)	2.6
VT/VF	33 (18.86)	150 (29.53)	25.0	33 (19.2)	52 (30.2)	25.8
AVB	36 (20.93)	97 (19.32)	4.0	36 (20.9)	37 (21.5)	1.4
AF	24 (13.71)	119 (23.43)	25.0	24 (14)	40 (23.3)	24.1
Cardiomyopathy, n (%)			18.0			
DCM	130 (74.29)	360 (70.87)		128 (74.4)	123 (71.5)	6.6
HCM	4 (2.29)	8 (1.57)		4 (2.3)	4 (2.3)	0.0
ARVC	1 (0.57)	1 (0.20)		1 (0.6)	0 (0)	9.8
RCM	4 (2.29)	26 (5.12)		4 (2.3)	5 (2.9)	3.6
Medication, n (%)						
ACE/ARB	146 (83.43)	415 (81.69)	5.0	143 (83.1)	141 (82)	3.1
β-blocker	155 (88.57)	447 (87.99)	2.0	153 (89)	152 (88.4)	1.8
Spiro	165 (94.29)	427 (84.06)	33.0	162 (94.2)	151 (87.8)	22.5
Digoxin	90 (51.43)	300 (59.06)	15.0	88 (51.2)	96 (55.8)	9.3
Diuretics	160 (91.43)	467 (91.93)	2.0	157 (91.3)	159 (92.4)	4.3
Statin	74 (42.29)	231 (45.47)	6.0	73 (42.4)	75 (43.6)	2.4
Amiodarone	21 (12.00)	97 (19.09)	20.0	20 (11.6)	32 (18.6)	19.6
Functional class, n (%)			29.0			
NYHA I	2 (1.18)	6 (1.21)		2 (1.2)	2 (1.2)	0.3
NYHA II	50 (29.59)	131 (26.46)		50 (30.1)	51 (30)	0.3
NYHA III	106 (62.72)	282 (56.97)		103 (62)	88 (51.8)	20.9
NYHA IV	11 (6.51)	76 (15.35)		11 (6.6)	29 (17.1)	32.7
LVEF (<35%), n (%)	127 (72.57)	413 (81.30)	21.0	126 (73.3)	131 (76.2)	6.7
CRT-D, n (%)	82 (47.13)	295 (58.07)	22.0	82 (47.7)	95 (55.2)	15.2
LVEDD, mm	68.09 ± 9.15	70.33 ± 9.94	23.0	68.01 ± 9.15	69.98 ± 10.30	20.2
BVP proportion, %	83.26 ± 34.91	82.49 ± 35.65	2.0	84.72 ± 33.41	78.12 ± 39.02	18.2
QRS duration, ms	167.90 ± 23.78	162.98 ± 23.73	21.0	167.90 ± 23.78	160.36 ± 23.32	32.0
NT-proBNP, pg/mL	1733.47 ± 1785.99	2253.39 ± 2568.71	24.0	1710.70 ± 1733.10	2027.64 ± 2917.72	13.2
eGFR, mL/min/1.73 m^2^	94.86 ± 39.23	101.04 ± 47.07	12.0	94.73 ± 39.08	98.21 ± 51.98	7.6

Values are given as mean ± SD or n (%) unless otherwise indicated. The standardized differences are reported as percentages; a difference of less than 10.0% indicates a relatively small imbalance. LOH: length of hospitalization; BMI: body mass index; LBBB: left bundle branch block; CAD: coronary artery disease; VT/VF: ventricular tachycardia/ventricular fibrillation; AVB: atrioventricular block; AF: atrial fibrillation; DCM: dilated cardiomyopathy; HCM: hypertrophic cardiomyopathy; ARVC: arrhythmogenic right ventricular cardiomyopathy; RCM: restrictive cardiomyopathy; ACEI/ARB: angiotensin converting enzyme inhibitor/angiotensin receptor blocker; Spiro: spironolactone; NYHA: New York Heart Association; LVEF: left ventricular ejection fraction; CRT-D: cardiac resynchronization therapy-defibrillator; LVEDD: left ventricular end-diastolic diameter; BVP: biventricular pacing; NT-proBNP: N-terminal prohormone of brain natriuretic peptide; eGFR: estimated glomerular filtration rate.

**Table 2:** Comparison of cardiac function at follow-up and outcomes between matched groups using GenMatch matching.

Variables	Standard LOH (n = 689)	Prolonged LOH (n = 689)	*p* Value
LVEF, (%)	41.19 ± 10.85	38.94 ± 11.71	0.001
Change in LVEF, (%)	10.70 ± 11.80	9.67 ± 9.59	0.126
LVEDD, mm	62.73 ± 10.01	64.06 ± 11.65	0.049
Change in LVEDD, mm	−5.33 ± 8.26	−6.22 ± 8.32	0.086
NT-proBNP, pg/mL	1121.36 ± 1360.37	1382.70 ± 1583.36	0.006
Change in NT-proBNP, pg/mL	−753.00 ± 1408.75	−728.86 ± 1918.07	0.823
QRS duration, ms	144.25 ± 16.96	143.55 ± 19.25	0.483
Change in QRS duration, ms	−25.47 ± 28.24	−20.76 ± 25.52	0.001
All-cause death, n (%)	148 (21.7)	194 (28.2)	0.006
HF hospitalization, n (%)	131 (19.8)	220 (32.3)	<0.001

Values are given as mean ± SD or n (%) unless otherwise indicated. McNemar’s test was used for categorical variables, and Student’s *t*-test or paired sample test was used for continuous variables. LVEF: left ventricular ejection fraction; LVEDD: left ventricular end-diastolic diameter; NT-proBNP: N-terminal prohormone of brain natriuretic peptide; HF: heart failure.

## Data Availability

The raw data supporting the conclusions of this article will be made available by the authors, without undue reservation.

## Supplementary Materials

The following supporting information can be downloaded at: https://www.mdpi.com/article/10.3390/jcdd9100354/s1, File S1: The corresponding propensity scores for each matched pair of patients.

## Abbreviations

LOH: length of hospitalization; HF: heart failure; GenMatch: Genetic Matching; BMI: body mass index; LBBB: left bundle branch block; CAD: coronary artery disease; VT/VF: ventricular tachycardia/ventricular fibrillation; AVB: atrioventricular block; AF: atrial fibrillation; DCM: dilated cardiomyopathy; HCM: hypertrophic cardiomyopathy; ARVC: arrhythmogenic right ventricular cardiomyopathy; RCM: restrictive cardiomyopathy; ACEI/ARB: angiotensin converting enzyme inhibitor/angiotensin receptor blocker; Spiro: spironolactone; NYHA: New York Heart Association; LVEF: left ventricular ejection fraction; CRT-D: cardiac resynchronization therapy-defibrillator; LVEDD: left ventricular end-diastolic diameter; BVP: biventricular pacing; NT-proBNP: N-terminal prohormone of brain natriuretic peptide; eGFR: estimated glomerular filtration rate; HR: hazard ratio; CI: confidence interval.
